# Using Text Messaging, Social Media, and Interviews to Understand What Pregnant Youth Think About Weight Gain During Pregnancy

**DOI:** 10.2196/11397

**Published:** 2019-04-01

**Authors:** Melissa DeJonckheere, Lauren P Nichols, VG Vinod Vydiswaran, Xinyan Zhao, Kevyn Collins-Thompson, Kenneth Resnicow, Tammy Chang

**Affiliations:** 1 Department of Family Medicine University of Michigan Ann Arbor, MI United States; 2 Department of Learning Health Sciences University of Michigan Ann Arbor, MI United States; 3 School of Information University of Michigan Ann Arbor, MI United States; 4 School of Public Health University of Michigan Ann Arbor, MI United States; 5 Institute for Healthcare Policy and Innovation University of Michigan Ann Arbor, MI United States

**Keywords:** methods, adolescents, weight gain, pregnancy, text messaging, social media, natural language processing

## Abstract

**Background:**

The majority of pregnant youth gain more weight than recommended by the National Academy of Medicine guidelines. Excess weight gain during pregnancy increases the risk of dangerous complications during delivery, including operative delivery and stillbirth, and contributes to the risk of long-term obesity in both mother and child. Little is known regarding youth’s perceptions of and knowledge about weight gain during pregnancy.

**Objective:**

The aim of this study was to describe the feasibility and acceptability of 3 novel data collection and analysis strategies for use with youth (social media posts, text message surveys, and semistructured interviews) to explore their experiences during pregnancy. The mixed-methods analysis included natural language processing and thematic analysis.

**Methods:**

To demonstrate the feasibility and acceptability of this novel approach, we used descriptive statistics and thematic qualitative analysis to characterize participation and engagement in the study.

**Results:**

Recruitment of 54 pregnant women aged between 16 and 24 years occurred from April 2016 to September 2016. All participants completed at least 1 phase of the study. Semistructured interviews had the highest rate of completion, yet all 3 strategies were feasible and acceptable to pregnant youth.

**Conclusions:**

This study has described a novel youth-centered strategy of triangulating 3 sources of mixed-methods data to gain a deeper understanding of a health behavior phenomenon among an at-risk population of youth.

## Introduction

### Background

Excess weight gain during pregnancy is a serious health concern affecting the majority of US youth who become pregnant [1-3], increasing the risk of dangerous complications during delivery and contributing to the risk of long-term obesity in both mother and child [4-7]. The risk of excess weight gain during pregnancy is especially high for overweight or obese youth, a population that continues to increase in the United States [1,2,8]. Given that more than one-third of US youth (aged between 12 and 19 years) are overweight or obese, and 71% of youth have had at least 1 sexual encounter by age 19 years, a large proportion of US overweight and obese youth are at risk [8-14]. The risk of excess weight gain is even more significant for low income and racial or ethnic minorities in the United States who have the highest rates of adolescent pregnancy and face significant barriers to nutrition and physical activity during pregnancy [15-19].

Healthy lifestyle interventions have been shown to promote healthy pregnancy weight gain in adults, which can decrease long- and short-term morbidity [20-26]. However, there is a significant gap in our understanding of youth’s perceptions of and knowledge about weight gain during pregnancy. Given this gap, current interventions cannot be adequately tailored to the distinct needs and preferences of pregnant youth and, consequently, may not be as effective [27].

### Conceptual Framework

**Figure 1:**
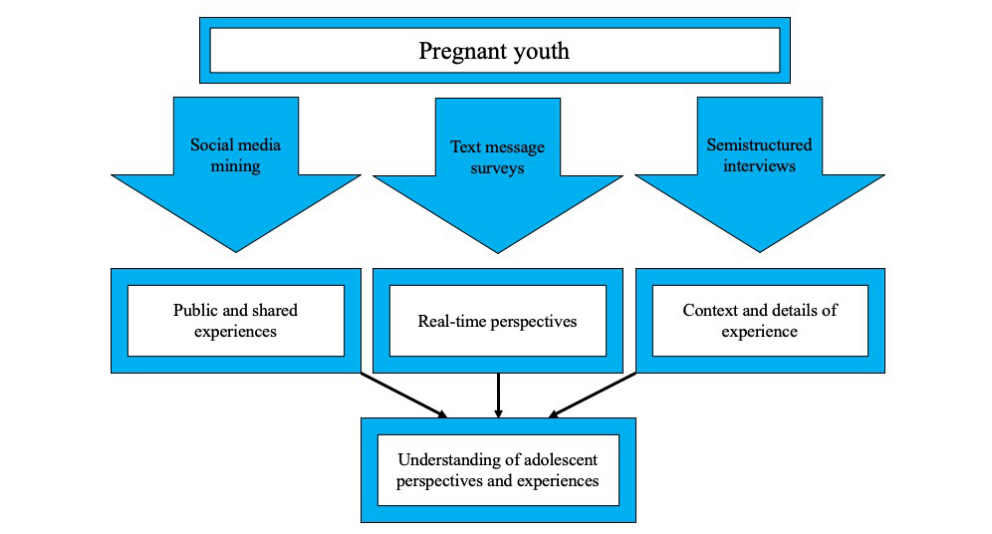
Conceptual framework of study design.

To understand the needs and preferences of pregnant youth, research approaches that appropriately respond to the unique circumstances of adolescence and young adulthood are needed. Many survey measures are not validated or tested among youth [28], whereas those that are are developed or fielded among school or college-based samples. To address these concerns, we have proposed a novel strategy of integrating 3 data sources that may be acceptable and accessible to high-risk youth to develop instruments or to tailor interventions: social media, text messaging, and interviews. Each data collection approach provides a unique perspective on youth behavior and experiences (see Figure 1).

Use of social media on the Web is pervasive and central to the way youth orient themselves to the world and others. With an increasing amount of time spent engaging on social media sites, much of the social and emotional development of youth is occurring on the Web. Regardless of race or socioeconomic status, nearly all youth (>80%) in the United States use social media on the Web with the majority spending several hours per day on the Web [29]. As a result, there is growing interest in social media content for influencing and understanding youth behavior [30-33]. Social media data are increasingly being used by adolescent health researchers, though studies predominantly are observational and reliant on public profiles [34-37]. Examining the social media posts of youth may improve our understanding of their beliefs and behaviors by capturing their *in vivo*, or organic, experiences that they choose to share with their social network. When analyzed using natural language processing (NLP), large amounts of text data (eg, months of Web posts) can be quickly processed and interpreted.

Text messaging is used by nearly all youth aged between 18 and 24 (97%) years and is central to their communication and relational experiences. Text messaging is the preferred form of communication among some subgroups of the population owing to its convenience and efficiency, including African American and Latino youth, individuals with lower educational attainment, and those with a lower income level [29,38,39]. When used in research, text messaging is the preferred mode of data collection among low-income communities [40,41] and results in more accurate data than other types of data collection such as in-person interviews, questionnaires, and in-person observation [42,43]. Although predominantly used to deliver information in research (eg, educational or behavioral interventions), studies are beginning to demonstrate the feasibility and acceptability of text messaging for data collection in youth populations [44]. Data collection via text messaging will allow researchers to gather real-time, immediate feedback rather than relying on recall.

Although both social media and text messaging capture essential aspects of youth experiences, it may be difficult to probe for a deeper understanding of participant experiences using these data collection tools. Qualitative semistructured interviews can complement social media and text messaging data by providing more in-depth context on the thoughts and beliefs of youth.

Interviews allow researchers to probe for more detail when key topics are mentioned, ask for clarification, or explore divergent responses. Interviews have frequently been used with high-risk and marginalized populations, including minority and immigrant youth [45] and pregnant youth [46]. To our knowledge, this traditional method of data collection has not been used with pregnant youth to elicit their understanding or perspectives related to weight gain during pregnancy. Together, social media, text messaging, and semistructured interviews can provide different perspectives into the experiences of youth.

### Objectives

In this study, we have presented an integrated methodological approach to understand youth perspectives and behavior related to weight gain during pregnancy. For the purpose of this study, we use *youth* to indicate adolescents and emerging adults within the 15 to 24 age range. We integrated multiple sources of data (social media posts, text message surveys, and semistructured interviews) to capture the complexities of youth experiences more fully. The ultimate goal of this study was to examine the feasibility and acceptability of integrating multiple youth-centered methods to inform the development of interventions to reduce excess weight gain. Our guiding research question was as follows: How do pregnant youth engage with social media mining, text message surveys, and semistructured interviews to provide insight into their experiences with weight gain during pregnancy?

## Methods

### Overview

The Healthy Pregnancy Project was designed to understand the perspectives of low-income youth during pregnancy to develop a tailored intervention to reduce excessive weight gain. To develop a tailored intervention that is responsive to the needs of pregnant youth, we collected information via youth-friendly methods including mining social media and text message surveys. The Institutional Review Board of the University of Michigan Medical School approved this study (HUM00104989).

### Setting

Participants were recruited from 2 low-income urban primary care clinics in Southeastern Michigan. These 2 clinics serve over 70% of the pregnant youth in the county.

### Participants

Eligible participants were youth aged between 14 and 24 years with a healthy singleton pregnancy at the time of recruitment, a gestational age of less than 24 weeks, an ability to read and speak English, and a cell phone with text messaging capabilities.

### Recruitment

**Figure 2:**
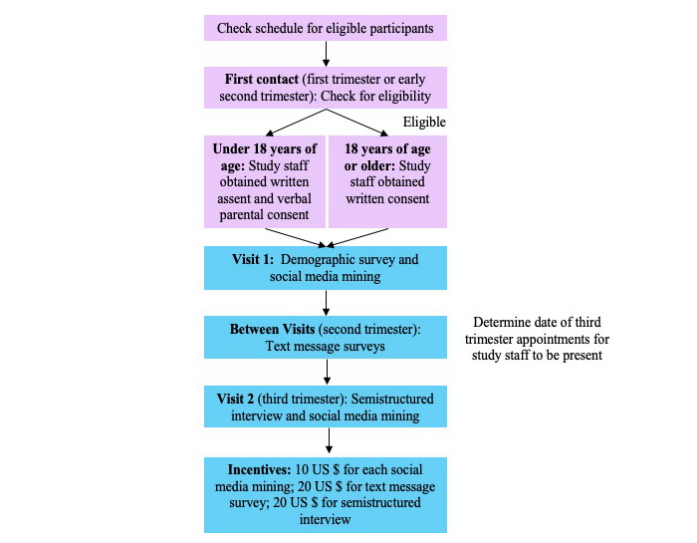
Recruitment and study process.

Study staff reviewed clinic schedules and electronic medical records daily to identify participants that appeared to meet the inclusion criteria (eg, age, singleton pregnancy, and gestational age). Participants were recruited before or after regular medical appointments at either of the 2 clinics (see Figure 2 for recruitment and study process) to decrease the burden for both patients and providers. Study staff confirmed eligibility, explained the study requirements, and answered questions from participants. Participants were informed that the study was called *Healthy Pregnancy Project* and the purpose was to learn more about young adults’ beliefs and knowledge about weight gain during pregnancy.

Written consent or assent was obtained for all participants. If the participant was aged under 18 years, written assent was obtained from the participant and verbal consent was obtained from the participants' parent or guardian. As minors may seek care without the permission of their parents or guardians at these clinics, pregnant youth who wished to maintain confidentiality from their parents or guardians regarding their pregnancy were excluded from the study. If the youth required care from high-risk obstetrical providers during the course of their pregnancy, they were unenrolled but received incentives for all completed study components. All participants were able to opt out of any study component other than the demographic survey completed upon enrollment. For example, they were able to only participate in the text message survey or only social media mining.

### Healthy Pregnancy Project Data Collection

#### Visit 1 (First Trimester or Early Second Trimester): Recruitment Visit and Demographic Data

##### Demographic Survey

After being consented, participants completed a demographic survey that collected contact information (cell phone number), demographics (eg, race, education status, and family characteristics) and medical information (eg, body mass index and due date) on a tablet using Qualtrics Survey Software (Qualtrics, Provo, UT) (see Multimedia Appendix 1 for demographic survey items). Questions were adopted from validated surveys when available. Participants aged under 18 years were asked to complete an adolescent socioeconomic status measure [47], whereas participants over the age of 18 years were asked to report their annual household income and household size.

##### Social Media Mining

We mined participants’ social media data, to be analyzed via NLP, to elicit a more complete and unfiltered version of participant *in vivo* experiences. Participants were asked to consent to mining of their social media accounts at 2 points: during the recruitment visit (generally, first trimester) and during the third trimester. Study staff performed a single download of the text posted on Facebook (up to the past year), the text posted on Twitter (up to 3200 tweets), or the text and images posted on Instagram (up to 50 previous posts) at each time point. The downloaded file included a timestamp and the raw text from the original comments and posts. Participants logged into their accounts, and data were extracted using the corresponding platform’s query application programming interface (API). On the API platform, we sent requests to participants’ accounts for access to their posted messages. Only after participants authorized our requests could we obtain access to the data. When the data collection procedure was completed, participants turned the accounts into private status to prevent any incidental unauthorized data collection. After extraction, participants signed out of their accounts. Between visits and after completion of the study, the staff did not have access to participants’ social media accounts. To protect the anonymity of comments of others on the Web that may be observed during social media mining, only comments and posts made by consented participants were downloaded. We did not record *likes*, *shares*, or other information available within their social media content. No identifying data were recorded from nonconsented individuals.

Participants were reminded of future communications with the Healthy Pregnancy Project research staff: (1) text message surveys for 8 weeks and (2) a follow-up interview and social media mining during their third trimester. Participants provided a short message service–capable phone number to complete the text message surveys.

#### Between Visits

##### Text Message Surveys

We used text messages to ask questions about young pregnant women’s real-time experiences during pregnancy. On the basis of previous work with text message surveys [40,41], the surveys were designed to be low cognitive burden, to which participants could quickly respond. The questions were developed by the research team to be youth friendly by using a conversational tone, asking short closed- and open-ended questions, and including supportive and empowering messages. For example, “Pregnancy is a time of a lot of changes in your body. Tell us how you feel about these changes. We want to hear the good and the bad!” Questions probed for youth knowledge and behaviors around diet, exercise, body image, and health during pregnancy (see Multimedia Appendix 2 for text message survey items). Some messages included multipart questions on the same topic (eg, identifying unhealthy behaviors and then describing barriers). Participants began to receive text message surveys during the second trimester to align with the period in which many women gain the most weight when pregnant [48]. Therefore, participants began and ended text message surveys asynchronously, beginning at 20 weeks gestation and continuing weekly for 8 weeks.

The text message survey questions were sent through an automated secure texting platform that met the Health Insurance Portability and Accountability Act requirements. Participants responded directly to the questions via text message at their convenience. Responses were stored in a secure server until they were downloaded for analysis at completion of the study. Before the Week 1 survey, participants received a tutorial that explained the process, including how to skip questions and how to stop receiving messages at any time. Participants were sent each weekly poll up to 2 times if they did not fully complete the poll the first time. Weekly polls were also resent if study staff learned that the participant’s phone number changed.

#### Visit 2 (Third Trimester): Semistructured Interview and Social Media Mining

##### Semistructured Interview

Using a semistructured interview guide consisting of predominantly open-ended questions, we elicited the participants’ knowledge, beliefs, and perspectives regarding weight gain during pregnancy. Research staff regularly reviewed electronic medical records for enrolled participants to identify upcoming visits in the third trimester. The staff then contacted participants via phone the night before an appointment to confirm that the participant had time to meet for a 30-minute one-on-one interview before or after the scheduled visit. Research staff trained in qualitative interviewing (postdoctoral research fellow, project coordinator, and medical students) met with participants in a private exam room before or after their perinatal visit. The semistructured interview guide was designed to allow for follow-up and probing questions to clarify participant responses or add detail. Interviews were audio-recorded, and the audio files were uploaded to a secure Web folder.

The interview guide included the following domains: knowledge about eating and drinking during pregnancy, weight gain during pregnancy, barriers to healthy eating and exercise, and behaviors related to eating, cooking, and exercise (see Multimedia Appendix 3 for the interview guide).

Interviews also explored participant experiences with the text message surveys, including acceptability and preferences. Interviews were performed during the third trimester of pregnancy so that participants had more personal experiences with pregnancy-related weight gain and had an opportunity to complete all text message surveys. When available, interviewers used the mined social media data (see the Natural Language Processing of Social Media Data section) from the first visit and text message survey responses to supplement the interview guide. For example, interviews would reference posts made on social media related to their pregnancy, weight gain, eating, exercise, or body image and ask participants to expand upon their statements or explain any contradictions.

##### Social Media Mining

During this visit, participants with social media accounts logged into their accounts again to allow research staff to download the recent posts from Facebook, Twitter, or Instagram. Files were saved to a secure Web folder. Participants would then log off their accounts, and the study staff would no longer have access.

##### Chart Review

Participants’ electronic medical records were reviewed to verify gestational age, identify the date that the pregnancy was confirmed by medical personnel, and record weight gain.

##### Incentives

Participants were compensated for each completed study component for a total of up to US $60: US $10 for each social media mining; US $2 per week for text message survey responses; US $4 bonus for completing all 8 weeks of text message surveys; and US $20 for completing the semistructured interview during the third trimester.

### Healthy Pregnancy Project Data Analysis

#### Baseline Survey

After study completion, participant responses were downloaded from the data management software as a comma-separated value (CSV) file. We used descriptive statistics to describe participant demographics, including race, ethnicity, median household income, and educational attainment.

#### Text Message Surveys

Text message survey responses were downloaded from the texting platform as a CSV file. Data were analyzed using an inductive approach adapted from grounded theory principles. First, all open-ended survey responses were compiled and read by the research team to gain a holistic sense of the data. Second, 2 members of the research team applied descriptive codes to full text message responses or segments of text to represent the central concept. Finally, preliminary categories were created by grouping text message responses (or phrases from text messages) into groups of similar concepts. The descriptive codes and categories were compiled into a codebook that was used when analyzing subsequent transcripts. Subsequent text messages were independently analyzed using the emergent codebook. Using an iterative process, the research team met periodically to identify new codes, modify the codebook, and resolve differences. Codes were organized into themes. Response rates were also calculated to understand participant engagement with text messaging during pregnancy. Data management and analysis were supported by MAXQDA12 qualitative data analysis software (VERBI GmbH; Berlin, Germany).

#### Semistructured Interviews

Audio recordings of one-on-one interviews were transcribed professionally and reviewed by the research team for errors. Data analysis was conducted using a process similar to the strategy for analyzing the text message survey responses detailed above. During the initial analysis phase, preliminary descriptive codes were created inductively from phrases of similar meaning from the first 2 interviews. Descriptive codes were used to build a codebook that was modified as analysis progressed. Subsequent transcripts were analyzed line-by-line by at least 2 qualitative researchers using the emerging coding scheme. Coders met periodically to identify new codes and arbitrate differences and create a final coding scheme. After all transcripts were coded, the codes were condensed into categories that represented emergent themes. Data management and analysis were supported by MAXQDA12.

#### Natural Language Processing of Social Media Data

Social media data were analyzed through NLP approaches to understand youth knowledge, beliefs, and social norms regarding weight gain during pregnancy. NLP is an approach to text analysis that allows researchers to review large amounts of free-text data and can augment traditional qualitative data collection and analysis. First, we created a list of keywords and phrases related to the following domains: pregnancy, weight gain, eating, exercise, body image, and stress. We augmented these words with synonyms and related words from linguistic resources such as WordNet [49] and the Linguistic Inquire and Word Count [50]. For example, to search for content related to pregnancy, we included words related to pregnancy and baby. For content related to weight gain, we included weight, pounds, diet, eat, belly, tummy, skinny, and other common diet- and weight-specific words. We preprocessed and tokenized the text posts and normalized morphological variants (babies vs baby and pregnancy vs pregnant). We searched for this expanded list of words and phrases of interest in the collection of social media posts on the Web made by participants on Facebook, Instagram, or Twitter. The relevant posts were compiled for each participant and analyzed using a traditional qualitative approach. The content that was posted before the participant’s date of conception was separated from the content posted after the participant was pregnant to allow for comparison.

In addition, the results of the NLP analysis were used to augment data collection during the one-on-one interviews. The compiled lists of relevant responses (related to pregnancy, weight gain, eating, exercise, body image, and stress) were used in 3 primary ways: (1) to initiate participant responses about a particular topic; (2) to provide context for participants to expand on a particular topic; or (3) to validate previous responses with additional evidence.

### Assessment of Acceptability and Feasibility of Methods

We calculated response rates for each data collection strategy (social media mining, text message surveys, and interviews). During the semistructured interview, the final component of data collection, participants were asked questions related to the participation process. Participants reflected on their participation (or limited participation) in the text message surveys and social media mining. Responses were analyzed using a qualitative thematic approach, as described above, focusing on the Healthy Pregnancy Project process.

## Results

### Participants

In total, 54 participants were enrolled in the study; 5 participants were later withdrawn for having high-risk pregnancies (eg, premature delivery and short cervix) and 3 were excluded after changing medical providers. In total, 46 participants completed at least 1 data collection component (social media mining, text messaging, or interview) of the study (see Table 1 for participant demographics) and were included in the qualitative evaluation presented here.

### Social Media Mining

We mined social media data from at least 1 platform for 39 participants (85%; see Table 2). The majority of participants provided access to their social media data available on Facebook (n=33 in the first trimester and n=37 in the third trimester) rather than Instagram (n=7 in the first trimester and n=0 in the third trimester) or Twitter (n=2 in the first trimester and n=1 in the third trimester). Participants who did not complete this component of the study either did not have any social media account (n=2), could not remember the correct log in information (n=2 during the first trimester and n=1 during the third trimester), or were unable to provide data because the mining process failed when attempted by the study staff (n=4 during the first trimester and n=1 during the third trimester). In all, 2 participants declined to participate at both time points, and a reason was not provided.

The mined social media data were used in 2 ways. First, analyzed social media content was used in semistructured interviewing when available. Using this content as an elicitation tool added to semistructured interviews by prompting participants to provide greater context and depth in their responses, validating previous responses, finding new and missing themes, and supporting participants in discussing personal and sensitive topics.

**Table 1:** Participant demographics.

Demographics		Statistics
Age (years), mean (SD)		21.2 (2.2)
**Income (US $)**		
	Median household income	7200
**Race, n (%)**		
	Non-Hispanic black	22 (48)
	Non-Hispanic white	13 (28)
	Hispanic or Latino	5 (11)
	Mixed race	5 (11)
	Other	1 (2)
**Educational status, n (%)**		
	Some high school	12 (26)
	High school graduate	21 (46)
	Some postsecondary education	13 (28)

Second, social media content was compared before and after each participant’s conception date to identify changes in perspectives and behaviors following pregnancy. For example, Oram et al [51] examined youth perspectives toward substance use shared on Facebook in the year before pregnancy and during pregnancy. This analysis allowed us to gain insight into shifts in behavior as a result of pregnancy and opportunities to reduce substance use in this population.

### Text Message Surveys

In total, 34 of the participants (74%) responded to at least 1 week of survey questions. Response rates were over 50% for each of the 9 weeks (see Table 3). In all, 10 of 46 participants (24%) completed the text message surveys for all weeks, including the tutorial.

Text message data were used to understand recent and current thoughts, beliefs, and behaviors that youth were engaging in during pregnancy. The data provided quick snapshots into the daily experiences of the participants. Unexpectedly, some participants viewed the text message surveys as a form of informational and social support.

Participants were asked to reflect on the text message surveys as part of the semistructured interview. Thematic analysis revealed a variety of factors supporting engagement with the text message surveys (eg, convenience and ease of use, reminder of healthy behaviors, and social support) and a few barriers to engagement (eg, one-way communication, phone-related barriers, time, and schedule conflicts). Themes and representative quotes depicting the acceptability of text message surveys are summarized in Textbox 1.

**Table 2:** Social media mining response rate by platform.

Visit	Facebook mined, n (%)	Instagram mined, n (%)	Twitter mined, n (%)
Visit 1: first trimester	33 (72)	7 (15)	2 (4)
Visit 2: third trimester	37 (80)	0 (0)	1 (2)

**Table 3:** Response rate for text message surveys.

Timepoint	Response rate upon completion, n (%)
Tutorial	23 (50)
Week 1	25 (54)
Week 2	27 (59)
Week 3	25 (54)
Week 4	24 (52)
Week 5	24 (52)
Week 6	26 (57)
Week 7	24 (52)
Week 8	25 (54)
All weeks	10 (22)

**Themes and representative quotes related to the acceptability of text message surveys.**

**Factors supporting engagement:**

- Convenience and ease of use

I’m always on my phone, so when they popped up, it was easy to respond. It really wouldn’t have mattered what time you sent them, ‘cause I still would have responded.

Participant 56, age 19

I think that’s as easy as it can get with it being a text.

Participant 29, age 20

I think that was a brilliant idea because any other way would be like probably inconvenient for some people.

Participant 12, age 22

I did reply late to one of ‘em ‘cause I was at work and I can’t have my phone on the floor when I’m at work, so, I did have to wait ‘til I got off work to reply.

Participant 47, age 17

- Reminder of healthy behavior

I don’t mind getting a text. If it’s going to help me learn and I can share it with somebody else. I’m willing to always have new information on something. If I’m interested in it, I wouldn’t mind getting information on it.

Participant 53, age 24

It was actually something that was good when they asked me type of questions like that, because sometimes when they would text me like, well, what do you like to eat, I would text them back and tell them, and then I want to go get it because they reminded me of it. Like I want that right now!

Participant 56, age 19

I really wasn’t reflecting on my pregnancy that much. But when the questions came, I’m like, dang! I should really start thinking about this. Like, dang, do I even eat anything healthy?

Participant 47, age 17

- Social support

You have your doctors when you’re pregnant and that's when everybody’s telling you, you know, be healthy and they’re checking in on you and stuff like that. But other than that, it’s kind of like a dead zone for people being concerned about their health. So it’s probably good for people to have that little bit of a reminder every now and again that they want to know what you’re thinking about. People are always happy for you that you’re pregnant and they're congratulating you and stuff like that, and when you’re around, they, you know, they ask about it, but when you’re not, it’s like everybody got their own lives. They’re not really checking in on you as much as you might like need it.

Participant 28, age 20

It was nice, especially because I was going through that hard time with my mom and everything, so just the text messages just asking how I was doing every week was kind of like a positive thing to look forward to.

Participant 23, age 21

When you’re pregnant, you kinda lose a lot of friends, so getting that text is kinda reassuring. It’s not like a friend, but it’s, like, you know somebody is listening to you. Like, you can just tell somebody how you feel. It’s like a virtual friend. They just talk to you.

Participant 41, age 19

It was fun. Sometimes that was the only person that was texting me.

Participant 19, age 16

It kept me comforted. Some of their questions would make me feel like, “Oh, okay, maybe I'm just overreacting with this.” It was...I would say, it was helpful with the questions to keep coming. For me, it would keep my mind off of other things that worry me about the baby and be able to keep up with the questions and answers.

Participant 44, age 24

**Barriers to engagement:**

- One-way communication

I mean, I don’t really know how to put it. They weren’t bad. It was actually nice to have someone check up on you and see how your pregnancy’s going or whatever. I’m like, is this really the lady that I met up with, checking on me? Or is it like, not a spam, but a…something like that? Honestly, I didn’t think it was real. I mean, I knew it was real obviously because I knew I had signed up for it and I knew she told me I would be getting them. But is this really the lady? But I didn’t want that to be my response; is this the lady I met or is this, you know?

Participant 46, age 24

The text messages were kind of bothering because they were fake. I could’ve typed back 7QM and it would’ve sent me the same thing. I’m saying, I actually could’ve typed in anything. I could’ve handed [my son] the phone and typed in something... And it would’ve sent me the same thing. I didn’t like how it was robotic.

Participant 48, age 22

It would’ve been nice to be able to ask a question. I didn’t really understand it. I was trying to text it back, but I didn't get the response I was looking for, so I’m like, “Oh, okay, well maybe I just gotta wait until they text me back then. So that would be nice, if I would have questions.”

Participant 44, age 24

I was like is this a machine or can you really talk? I was like lonely, and I was tired. Like or when you asked that question about how are you feeling? I’d spill my heart out and it would say [in response], well, thank you for participating.

Participant 15, age 24

- Phone-related barriers

It’s like it’d come to my phone and then if I don’t have service in that area, I’ll reply back, and it don’t go.

Participant 18, age 23

I don’t have a phone, it just got cut off.

Participant 14, age 18

There was one time my phone was off and you guys had texted me and I couldn’t text back. I don’t think I was able to text for like a week.

Participant 48, age 22

- Time and schedule conflicts

I think I sometimes I would try to and then I would forget, cuz there’s sometimes I wanna say more than what I’m gonna say at that time that I have. I just forget to go back to it.

Participant 29, age 20

They came when I was asleep. I think they came every Friday, but I work afternoons. They did come before I went to work, but I would sleep until I go back to work the next day. Working a 10-hour shift is not easy.

Participant 46, age 24

### Semistructured Interviews

In total, 43 of the 46 participants (94%) were interviewed in person during their third trimester, before or after an appointment at the primary care clinic. Interviews ranged from 10:53 to 30:02 minutes, with an average length of 19:39 minutes. Participants discussed several domains including perceived stress; knowledge about weight gain, eating, and exercise; attitudes about weight gain, eating, and exercise; behaviors related to weight gain, eating, and exercise; and experiences while participating in the study. A full description of the qualitative results will be presented in subsequent studies.

Interview data were used to gain in-depth descriptions of participant beliefs and behaviors. Unlike in the text message surveys, the interviews included follow-up and clarifying questions. The interviewer was able to *follow* the participant to explore topics that emerged during the interview. An unexpected theme, for example, was the lack of social support experienced by many of the participants. Although participants were not explicitly asked about social support, they frequently discussed the role of others in their health behaviors during pregnancy. As a result, interviews played an essential role in identifying new themes that did not emerge from other data sources.

## Discussion

### Principal Findings

This study has demonstrated the potential integration of 3 data collection techniques with pregnant youth. Although independently providing one lens into youth perspectives, the integration of the 3 data sources provides a more complete picture of participant experiences.

Overall, 72% to 76% of participants provided their Facebook data, whereas only 2 participants provided their Twitter data and 7 participants provided Instagram posts. With all 3 platforms, participants who did not complete this component indicated that they did not use these social media platforms or that they could not remember their login information. Our participation rates are similar to the expected prevalence of Facebook use among this population [38]. Future studies that employ social media mining should consider the platforms that are typically used among their target population and how social media use may influence participation rates.

A majority of participants responded to at least 1 week of text message surveys, though engagement varied throughout the duration of the study. Text messaging may be a cost-effective means of communicating with youth participants to collect real-time data, inform intervention development, or investigate processes before, during, and after intervention studies. For the participants in this study, text message surveys were an acceptable modality that had an unexpected outcome of making youth feel supported and connected to others. Collecting survey data via text message allowed participants to control the time of day and space where they participated in the survey.

Text message surveys also present several challenges. Our findings revealed that many youths would have preferred 2-way communication and tailored messaging to engage them each week. Some participants found the language of the texts repetitive, whereas others indicated that the content felt too standardized. Our results also demonstrated that participants varied in their preferences for the frequency, timing, and language of text messages received. Tailoring the frequency, timing, and content of messages may increase acceptability and engagement for participants who were less responsive in the text message surveys, though that may limit the ability to scale text message surveys to larger populations owing to the amount of staff and resources required.

Despite participants having access to a cell phone at the time of enrollment, continued access and regular use of cell phones varied. Several participants reported that they did not participate in the text message surveys owing to a change in cell phone number, poor service, or cell phone plan limits on text messaging. Similar problems were observed with surveys administered via phone or through Web platforms, and innovative strategies to enroll and sustain participation among low-income communities should be addressed in future research [52].

The semistructured interviews had the highest participation rates among all components of the study. As the youth were interviewed before or after an existing, regularly scheduled perinatal visit with their primary care physician, we were able to reduce the time burden to participation. Interviews were conducted in a familiar setting, which may have helped in developing trust and encouraging participation [53]. As the research was supported by the 2 clinical sites, the medical staff served as gatekeepers [54] and facilitated interactions between the research staff and participants. Face-to-face contact may play an important role in engaging pregnant youth who are often balancing many responsibilities. In addition, semistructured interviews allowed for more in-depth exploration of study topics. Interviewers could ask follow-up questions, probe for more information, and use content from social media mining and text message surveys to elicit further details.

All 3 components of the study provided a different lens through which we were able to investigate gestational weight gain among pregnant youth. Through social media mining, we gained access to public beliefs and statements about pregnancy, weight gain, body image, and eating in the months when the women were pregnant. The text message surveys provided real-time descriptions of these same domains, allowing participants to reflect upon their recent experiences and express personal details or opinions they may not want to share with their social network. Finally, the semistructured interviews produced in-depth narratives of participant experiences over the course of the pregnancy. Interviews allowed the research team to ask follow-up and probing questions that clarified statements and added to our understanding of their lived experiences. Together, the combination of these 3 methods produced a more complete picture of weight gain during pregnancy among an at-risk population of low-income youth.

### Limitations and Future Directions

There were several limitations to this study. First, recruiting and retaining youth in research is difficult [21,55]. Pregnancy is also a turbulent and stressful time, which could make study components feel burdensome. The study population may seek care late into pregnancy, making it difficult to complete the study components before their due date. We tried to reduce the burden experienced by the participants by approaching them before or after their regularly scheduled clinic visits. In addition, we minimized the time spent with participants and aimed for all visits to last less than 30 minutes.

Second, using text messaging to collect survey data is a potential limitation. Study participants may have difficulty paying cell phone bills, which may result in their cell phone number changing or service ending during the study. This could result in them not receiving the text messaging questions part way into the study. To overcome this barrier, we provided all participants with a notecard that had our contact information so that they could call, text, or email if their phone number changed during the study. We were also unable to verify that the text message responses received were from the participant themselves or from someone using the participant’s phone.

Third, although no participants expressed this concern, potential future participants may have privacy concerns about answering sensitive questions over text message or providing access to their social media accounts. To address this concern, we ensured that the texting platform used for this study was usable and secure through extensive pilot testing with a similar population. Participants were allowed to consent to individual components of the study, so interviews could be done without either text message surveys or social media mining.

Finally, some participants described the text message surveys as a source of social support. As the intention of our study was to collect data about participant experiences and not to intervene, there is potential bias in the responses of the pregnant youth. Studies employing text message surveys with youth will want to consider the potential influence that text messaging may have on participant responses.

### Conclusions

Despite these limitations, we demonstrated the feasibility and acceptability of these data collection methods among at-risk pregnant teens in low-income settings. Moderate response rates to the text message surveys and willingness to provide access to social media accounts indicate that participants were comfortable sharing data through these developing modalities.

## Appendix

Multimedia Appendix 1 MyVoice demographic survey completed upon enrollment.

Multimedia Appendix 2 Text message surveys by week.

Multimedia Appendix 3 Semistructured interview guide.

## Abbreviations

API: application programming interface
CSV: comma-separated value
NLP: natural language processing
