# Supplementary material for: Using Text Messaging, Social Media, and Interviews to Understand What Pregnant Youth Think About Weight Gain During Pregnancy
Source: JMIR Form Res. 2019 Apr 1;3(2):e11397. doi: 10.2196/11397 (PMC6462892; doi:10.2196/11397)
Supplement: Multimedia Appendix 1 [file formative_v3i2e11397_app1.pdf]

Domain	Question	Response options
**Contact**	What is your cell phone number?	Open-ended
**Height**	What is your height?	Open-ended
**Weight**	What is your weight?	Open-ended
**Gestational age**	What is your due date? (Please type in the month and day if you can remember!)	Open-ended
**Family status**	Who do you live with?	Mom
		Dad
		Brother(s)
		Sister(s)
		Aunt(s)
		Uncle(s)
		Cousin(s)
		Grandparent(s)
	Are your parents:	Married

		Together but not married
		Divorced
		Not together, never married
		Other (empty text box to explain)
**Educational attainment**	Are you enrolled in school?	Yes
		No
	If Yes to question above:<br><br>What grade are you in?	Open-ended
**Race/ethnicity**	What is your race? Check all that apply.	Black
		White
		Asian
		Native Hawaiian
		Mixed Race. Please describe.
		Other. Please describe.
	Are you Hispanic or Latino?	Yes
		No

**Socioeconomic status-adolescent** (received by participants under 18 years of age at the time of enrollment)	Does your family own a car?	Yes
		No
	Does your family own two cars?	Yes
		No
	Does your family own their home?	Yes
		No
		I don't know
	Does your family own a computer?	Yes
		No
	Do you have the option of free or reduced-price lunch at school?	Yes
		No
**Socioeconomic status-adult** (received by participants 18 years and older at the time of enrollment)	What is your annual household income? (Total amount of everyone in your household)?	Open-ended
