# Supplementary material for: Using Text Messaging, Social Media, and Interviews to Understand What Pregnant Youth Think About Weight Gain During Pregnancy
Source: JMIR Form Res. 2019 Apr 1;3(2):e11397. doi: 10.2196/11397 (PMC6462892; doi:10.2196/11397)
Supplement: Multimedia Appendix 2 [file formative_v3i2e11397_app2.pdf]

Time-point	Message sent to participants
**Enrollment**	Hi there! We're so happy you agreed to participate in the Healthy Pregnancy Project! First, let's go over some survey guidelines, okay? Text "next" to continue.
	For each question, you have to send something in order to get the next text. So even if you don't have an answer, send something! (Remember to text next to continue).
	If there's a question you don't feel comfortable answering, just text "skip" to skip, or text "STOP" to stop participating in the Healthy Pregnancy Project text surveys completely. (Remember to text next to continue).
	Remember all of your responses are confidential and analyzed by a computer, so please call 866-812-8632 if you have any questions or concerns. (Remember to text next to continue).
	You have the entire week to answer the questions. Make sure you answer them all to get your credit! (Remember to text next to continue).
**Week 1**	Hi, this is the Healthy Pregnancy Project! Congratulations on making it to the second trimester! How are you doing today?
	This is the first set of questions you will get to help us better understand your pregnancy experience. Remember there are no right or wrong answers! Type OK when you are ready to start.
	Let’s start simple. What 5 foods do you eat the most? Feel free to list more or less!
	Thank you! 7 more weeks left!
**Week 2**	Hey again from the Healthy Pregnancy Project! How are you doing?
	Last week we asked you about the foods you eat. This week we are curious about what you drink. Are you ready for the next question?

	What do you drink on a typical day, in order from most to least?
	Thank you for your thoughts! 6 more weeks left!
**Week 3**	Hello from the Healthy Pregnancy Project! How has your week been so far?
	This week we want to hear your thoughts on pregnancy cravings. Do you have them? How do you deal with them?
	Thank you for sharing that. That's it for this week, 5 more weeks to go!
**Week 4**	Hello from the Healthy Pregnancy Project! How are you feeling this week?
	This week we would like to hear your thoughts on exercise during pregnancy. Have you been exercising? Tell us what you have been doing and why or why not!
	Thanks for your thoughts! 4 more weeks to go!
**Week 5**	Hello mama-to-be! This is the Healthy Pregnancy Project again! What would you say are the healthiest things you are doing during your pregnancy?
	You're done for the week. Thank you! 3 more weeks to go!
**Week 6**	Hi! This is the Healthy Pregnancy Project! How has your week been?
	We know it’s hard to do everything right when you are pregnant. What do you think you could do to be healthier while pregnant? What's keeping you from doing it?
	Thank you for your thoughts! 2 more weeks to go!
**Week 7**	Hey there! You're past the halfway mark of your pregnancy! How does it feel?
	Many pregnant women have a lot of questions by this time in their pregnancy. What kinds of questions do you have about staying healthy during pregnancy?
	Where do you get information about staying healthy during pregnancy? Why do you use this resource?
	Thank you for sharing! Just one more week to go!

**Week 8**	Congrats! This is the last set of text message questions you will get. Thank you for sharing your thoughts! How's it going this week?
	Pregnancy is a time of a lot of changes in your body. Tell us how you feel about these changes. We want to hear the good and the bad!
	Thank you for participating! We hope to hear more of your thoughts when we talk in person. You will get your $20 incentive then. If you haven't scheduled a time, we will be in touch soon!
