# Supplementary material for: Using Text Messaging, Social Media, and Interviews to Understand What Pregnant Youth Think About Weight Gain During Pregnancy
Source: JMIR Form Res. 2019 Apr 1;3(2):e11397. doi: 10.2196/11397 (PMC6462892; doi:10.2196/11397)
Supplement: Multimedia Appendix 3 [file formative_v3i2e11397_app3.pdf]

Domain	Example Questions
General pregnancy questions	Tell me about how your pregnancy has been so far.
	Things that have been hard?
	Things that have been surprising?
	Anything that has worried you?
Diet during pregnancy	What are your thoughts about what you should **eat and drink** during pregnancy?
	What is healthy or unhealthy? Why?
	Tell us about any cravings, or changes in food preferences you have had.
	What have people told you about what to eat during pregnancy? Who? Who do you trust?
	What prevents you from eating healthy?
	Is there anything you would change about what you have been eating during pregnancy?
Cooking during pregnancy	Do you know how to cook? Who taught you?
	Who does the majority of cooking in your household?
	Do you cook most of your meals, eat out for most of your meals or somewhere in between?
Exercise during pregnancy	What are your thoughts about what you should do for **exercise** during pregnancy?
	What is healthy or unhealthy? Why?
	What is your physical activity like? Tell me about how you make these choices.

	What have people told you? Who? Who do you trust?
	What, if anything, worries you about exercising during pregnancy?
	What, if anything, would you change about what your exercise during pregnancy?
Weight gain during pregnancy	How much weight is someone like you supposed to gain during pregnancy? _____ (number of pounds)
	How much is too much weight to gain during pregnancy? _____ (number of pounds)
	How much have you gained? How much do you want to gain?
	How do you feel about how much weight you have gained?
	Is there anything that worries you about weight gain during pregnancy?
	Your body changes a lot during pregnancy. Has it changed how attractive you feel? Tell us more!
Text message acceptability	What did you think about the text message surveys?
	What did you like about participating?
	(If participant didn't respond to all questions)<br><br>What kept you from answering the rest of the text message surveys?

	What types of changes would you recommend to make it more convenient to answer the text message surveys?
